# GPT-4o vs. Human Candidates: Performance Analysis in the Polish Final Dentistry Examination

**DOI:** 10.7759/cureus.68813

**Published:** 2024-09-06

**Authors:** Aleksander Jaworski, Dawid Jasiński, Barbara Sławińska, Zuzanna Błecha, Wojciech Jaworski, Maja Kruplewicz, Natalia Jasińska, Oliwia Sysło, Ada Latkowska, Magdalena Jung

**Affiliations:** 1 Department of Plastic Surgery, Specialist Medical Center, Polanica-Zdrój, POL; 2 Department of Medicine, Prof. K. Gibiński University Clinical Center of the Medical University of Silesia in Katowice, Katowice, POL; 3 Department of Medicine, Medical University of Silesia in Katowice, Katowice, POL; 4 Department of Children’s Developmental Defects Surgery and Traumatology SUM (Medical University of Silesia) Scientific Club, Medical University of Silesia in Katowice, Zabrze, POL; 5 Department of Dentistry, Medical University of Silesia in Katowice, Zabrze, POL; 6 Department of Cybernetics, Military University of Technology, Warsaw, POL; 7 Department of Medicine, Academy of Silesia, Katowice, POL; 8 Department of Medicine, Wroclaw Medical University, Wrocław, POL; 9 Department of Dermatology, University Clinical Hospital in Opole, Opole, POL

**Keywords:** artificial intelligence, chatgpt, dentistry final medical examination, machine learning, medical education, medical professionals

## Abstract

Background

This study aims to evaluate the performance of OpenAI's GPT-4o in the Polish Final Dentistry Examination (LDEK) and compare it with human candidates' results. The LDEK is a standardized test essential for dental graduates in Poland to obtain their professional license. With artificial intelligence (AI) becoming increasingly integrated into medical and dental education, it is important to assess AI's capabilities in such high-stakes examinations.

Materials and methods

The study was conducted from August 1 to August 15, 2024, using the Spring 2023 LDEK exam. The exam comprised 200 multiple-choice questions, each with one correct answer among five options. Questions spanned various dental disciplines, including Conservative Dentistry with Endodontics, Pediatric Dentistry, Dental Surgery, Prosthetic Dentistry, Periodontology, Orthodontics, Emergency Medicine, Bioethics and Medical Law, Medical Certification, and Public Health. The exam organizers withdrew one question. GPT-4o was tested on these questions without access to the publicly available question bank. The AI model's responses were recorded, and each answer's confidence level was assessed. Correct answers were determined based on the official key provided by the Center for Medical Education (CEM) in Łódź, Poland. Statistical analyses, including Pearson's chi-square test and the Mann-Whitney U test, were performed to evaluate the accuracy and confidence of ChatGPT's answers across different dental fields.

Results

GPT-4o correctly answered 141 out of 199 valid questions (70.85%) and incorrectly answered 58 (29.15%). The AI performed better in fields like Conservative Dentistry with Endodontics (71.74%) and Prosthetic Dentistry (80%) but showed lower accuracy in Pediatric Dentistry (62.07%) and Orthodontics (52.63%). A statistically significant difference was observed between ChatGPT's performance on clinical case-based questions (36.36% accuracy) and other factual questions (72.87% accuracy), with a p-value of 0.025. Confidence levels also varied significantly between correct and incorrect answers, with a p-value of 0.0208.

Conclusions

GPT-4o's performance in the LDEK suggests it has potential as a supplementary educational tool in dentistry. However, the AI's limited clinical reasoning abilities, especially in complex scenarios, reveal a substantial gap between AI and human expertise. While ChatGPT demonstrates strong performance in factual recall, it cannot yet match the critical thinking and clinical judgment exhibited by human candidates.

## Introduction

Artificial intelligence (AI) has become a transformative force in many sectors, with healthcare being one of the most impacted [1]. AI applications range from diagnostic tools that aid in identifying diseases to educational platforms that assist in training healthcare professionals [2]. Among these applications, OpenAI's ChatGPT, a large language model, has gained attention for its ability to generate human-like text based on vast amounts of input data. ChatGPT is based on deep learning and natural language processing [3]. It quickly gained popularity after its launch in November 2022 and now has 180 million registered users [4]. ChatGPT's rapid growth outpaced platforms like Instagram, Facebook, and Twitter, making it one of the fastest-growing apps, second only to Threads [5]. Several generations have been released, including the first generative pre-trained transformer model in 2018, GPT-2 in 2019, GPT-3 in 2020, followed by the latest arrival of GPT-4 in 2023 [6]. The newest version, GPT-4.0, has arrived in various editions, such as basic GPT-4.0, GPT-4o, and GPT-4o mini. The “o” in GPT-4o stands for “Omni,” representing the model’s capability to accept various forms of input, including text, image, audio, and video, surpassing the capabilities of other existing models. Models from version 4 are not available for free, but since the introduction of version 4o, each user can ask a limited number of questions before reverting to version 3.5. Thus, to a limited extent, every user can test its functionality.

We conducted a study aimed at comparing the performance of ChatGPT 3.5 and 4o with medical professionals, such as fifth and sixth-year medical students and junior medical doctors, in the Polish Final Medical Examination (LEK). Although ChatGPT 3.5 did not pass the exam, we demonstrated that GPT-4o is capable of doing so without prior memorization of the question bank, achieving a 77.55% score - similar to the average human test-takers [7]. Given the increasing integration of AI in medical education, it is crucial to assess how AI tools like ChatGPT perform in high-stakes examinations compared to human candidates. The implications of such comparisons are significant, as they could inform the future use of AI in medical and dental training and assessment, potentially leading to new educational paradigms. The use of AI could assist in searching for information and theoretical details used during dental practice, developing dental treatment plans that take into account the patient's condition, and creating preventive care schemes that could later be shared with patients based on their individual clinical status, contributing to the advancement of personalized medicine. The capabilities of different ChatGPT versions in taking medical exams have been examined not only by us, referring to LEK, but also in fields such as allergology [8], radiology [9], dermatology [10], nuclear medicine [11], and cardiology [12]. Kung et al. reported the rising accuracy of ChatGPT, which approaches or exceeds the passing threshold for the USMLE exam [13]. However, these studies are elaborate and focus mostly on ChatGPT 3.5 performance. We anticipate that due to the variable and developmental nature of AI, it is crucial to evaluate the latest version of AI achievements.

The current study focuses on evaluating the performance of the most advanced version of ChatGPT, GPT-4o, in the Polish Final Dentistry Examination (LDEK), which is a critical standardized test required for dental graduates in Poland to obtain their professional license. The LDEK evaluates a wide range of dental knowledge and clinical skills, making it a rigorous test of both factual recall and clinical reasoning. The exam structure includes a division of questions into different fields of dentistry; however, there is no specified number of scenario-based questions for each of these fields. Additionally, this study compares ChatGPT's performance on the LDEK with human candidates' scores, providing a broader context for understanding AI's current capabilities and limitations in professional examinations.

## Materials and methods

The study was conducted between August 1 and August 15, 2024. It involved analyzing the Spring 2023 edition of the LDEK, which was randomly selected from previous exams in the question database of the Center for Medical Education (Centrum Egzaminów Medycznych, CEM) in Łódź, Poland. The selected exam initially consisted of 200 multiple-choice questions, each with one correct answer among five distractors, chosen using a random number generator. One question was withdrawn by CEM due to lacking a clear correct answer or being inconsistent with current medical knowledge.

To ensure a comprehensive analysis of all questions, they were divided into those related to "clinical cases," where the correct answer had to be chosen based on the clinical description of a specific patient, and "other" questions related to general medical knowledge. Additionally, all questions were categorized by CEM according to dental fields in line with the LDEK division, i.e., questions from Conservative Dentistry with Endodontics (46 questions), Pediatric Dentistry (29 questions), Dental Surgery (25 questions), Prosthetic Dentistry (25 questions), Periodontology (20 questions), Orthodontics (19 questions), Emergency Medicine (10 questions), Bioethics and Medical Law (10 questions), Medical Certification (7 questions), and Public Health (eight questions). Two independent researchers conducted the categorization, which was later reviewed and accepted by a third independent researcher. Due to the multicenter nature of the study, authors cooperated via remote communication methods, such as Microsoft Teams, Zoom, Facebook Messenger, emails, and Google Docs. All sections of the study prepared by the individual groups were reviewed by the other authors, allowing each researcher to contribute to every part of the study using the aforementioned remote communication methods.

Data collection and analysis

Before introducing ChatGPT to the questions, it was familiarized with the exam regulations, including information about the number of questions, the number of possible answers, and the number of correct answers. Furthermore, after each question was input into the model, ChatGPT was asked, "On a scale of one to five, how confident did you feel about the question?" This question aimed to gauge ChatGPT's confidence level in selecting answers. ChatGPT could respond to this confidence question as follows: one - unsure, two - not very sure, three - almost sure, four - very sure, five - completely sure. All questions were input into ChatGPT, and all interactions with it were documented. To maintain consistency with the content of the LDEK exam questions, the entire interaction with ChatGPT was conducted in Polish. The study used GPT-4o.

Statistical analysis

The results obtained from ChatGPT were compared with the correct answers recognized by CEM in Łódź. The assessment of ChatGPT's effectiveness involved determining the percentage of correct answers provided by ChatGPT (divided according to medical fields). ChatGPT's confidence in giving both correct and incorrect answers was also analyzed.

Pearson's chi-square test was used to assess the relationship (significance) between the distribution of correct and incorrect answers, question type, and other qualitative variables. The STATISTICA program (StatSoft, Tulsa, OK) was used for statistical analysis. Additionally, to compare the confidence level between correct and incorrect answers, the Mann-Whitney U test was applied. p-Values less than 0.05 were considered statistically significant.

## Results

**Figure 1:**
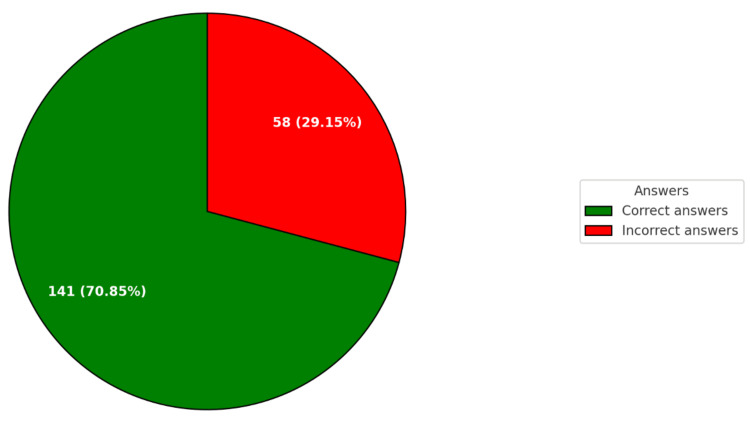
General summary of GPT-4o results.

GPT-4o provided correct answers to 141 questions (70.85%) and incorrect answers to 58 questions (29.15%) (Figure 1).

Among the 11 Clinical Cases questions, it correctly answered four questions (36.36%) and incorrectly answered seven (63.64%). In contrast, for the Other category, it correctly answered 137 questions (72.87%) and incorrectly answered 51 questions (27.13%). The difference in performance between the Clinical Cases and Other categories was statistically significant, with a p-value of 0.025 (Table 1). 

**Table 1:** GPT-4o results in clinical cases and other

Type of question	Correct answers, n (%)	Incorrect answers, n (%)	p-Value
Clinical cases	4 (36.36)	7 (63.64)	p = 0.025
Other	137 (72.87)	51 (27.13)	

The performance of GPT-4o across various fields of dentistry is available in Table 2. The p-value for the chi-square test is 0.0264, indicating a statistically significant difference in the number of correct and incorrect responses across the various categories of dentistry (Table 2). 

**Table 2:** GPT-4o results in specific medical fields

Medical field	Correct answers, n (%)	Incorrect answers, n (%)	p-Value
Conservative dentistry with endodontics	33 (71.74)	13 (28.26)	p = 0.0264
Pediatric dentistry	18 (62.07)	11 (37.93)	
Dental surgery	16 (64)	9 (36)	
Prosthetic dentistry	20 (80)	5 (20)	
Periodontology	11 (55)	9 (45)	
Orthodontics	10 (52.63)	9 (47.37)	
Emergency medicine	10 (100)	0 (0)	
Bioethics and medical law	8 (80)	2 (20)	
Medical certification	7 (100)	0 (0)	
Public health	8 (100)	0 (0)	

**Figure 2:**
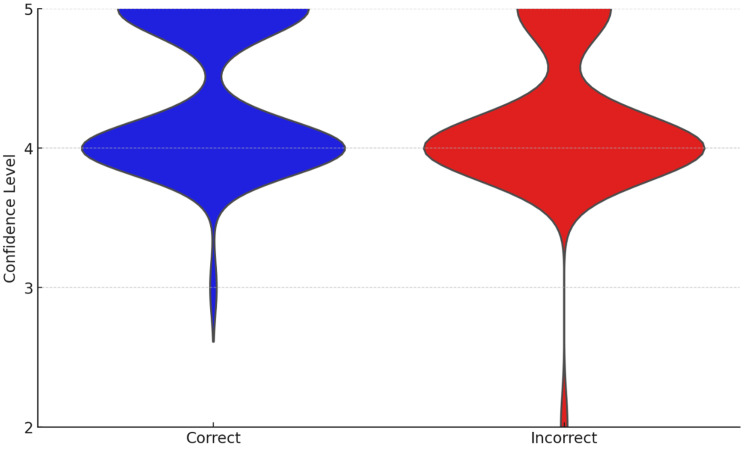
Comparison of confidence level distribution for correct and incorrect answers of GPT-4o.

In the Mann-Whitney test analyzing the confidence in answering questions both correctly and incorrectly, the p-value was 0.0208, indicating a significant difference in confidence (Figure 2).

## Discussion

A total of 1,109 candidates took the Spring Dental Medical Final Examination, a slight increase compared to the Autumn 2022 session but nearly 200 fewer than the Spring of the previous year, when 1,293 candidates participated. Unfortunately, 87 candidates failed to meet the passing threshold of 56%. The average score was 149.9 points (75.32%), which is over 5 points lower than in the previous two editions of the exam.

Among the universities whose students and graduates achieved the highest average scores, the top spot was claimed by the Collegium Medicum of Jagiellonian University, with an average score of 155.5 points (78.14%). In contrast, the lowest performance was observed among the representatives of the Medical University of Łódź, with an average score of 146.44 points (73.59%); notably, 23 of its candidates failed the exam, accounting for 26% of all those who did not pass [14]. Simultaneously, the best individual score was achieved by a representative of the same Medical University, with 189 points (94.97%) [15]. Graduates who completed their studies no more than two years ago performed significantly better on the exam, achieving an average score of 152.2 points (76.48%), compared to their more experienced peers, who averaged only 139.92 points (70.31%). Notably, among the 736 candidates taking the exam for the first time, 52 failed to pass [14]. This situation may stem from final-year students treating the exam as a practice opportunity before their official attempt post-graduation.

Our analysis revealed significant variations in ChatGPT's performance across different dental disciplines. In fields such as conservative dentistry and endodontics, GPT-4o demonstrated a level of accuracy comparable to the average human candidates. For instance, ChatGPT answered a substantial number of questions correctly, aligning with general human performance trends. However, in more clinically demanding areas like pediatric dentistry and oral surgery, the AI's accuracy decreased, suggesting it struggled with questions requiring applied clinical reasoning. While ChatGPT's overall performance was respectable, it fell short compared to the highest human scores, which reached up to 94.97%. GPT-4o’s performance was nearly 25% lower, highlighting a significant gap between AI and top-performing human candidates. Notably, ChatGPT achieved a perfect score in three specific categories, showcasing its potential in certain areas.

In contrast to medical students and junior doctors, who require high scores on the LEK to pursue specialty training, dentistry students and junior dentists do not face the same pressures. The lower percentage of young dentists pursuing specialty programs in Poland indicates a preference for private courses over formal residency programs. These private courses, though expensive, are seen as more accessible and practical by many junior dentists, who claim they offer superior training compared to traditional residency programs.

The average score for the Spring 2023 LEK was around 83.45%, with the highest score reaching 98.97%. Notably, 246 candidates scored below the passing threshold of 56%, meaning 97.11% (8,237) of the candidates passed [16]. This highlights a clear distinction between LDEK and LEK takers. Although direct comparison is challenging due to differences in the exams, the data suggest that medical students and junior doctors are more motivated to achieve high scores on the LEK compared to dentistry students and junior dentists on the LDEK. The medical career of a graduate in the field of medicine primarily depends on the specialization they obtain, which largely determines their future earnings and position among doctors. Admission to the chosen specialization is determined by the result of the LEK, which motivates students to prepare thoroughly for it. However, in the case of dental students, having a specialization is not as crucial for their career and earnings. As a result, a high score on the LDEK is not as important to them. Consequently, their preparation for this exam is not focused on achieving a high score but rather on simply passing the exam, with the passing threshold, as in the case of LEK, set at 56%.

GPT-4o's score, being 15% above the 56% threshold required to pass and slightly below the average human score, is impressive given that it did not have prior exposure to the exam's question bank. Historically, human test-takers also performed lower before the era of publicly available question banks [17]. Future iterations of ChatGPT could potentially surpass both the current version and human test-takers. The consistently high scores achieved by human candidates in the LDEK suggest that human cognitive abilities, particularly in critical thinking and clinical judgment, still surpass current AI capabilities. The familiarity with the question bank provides human candidates with an advantage that AI lacks.

These results indicate that while AI, such as ChatGPT, shows promising progress, it remains a complementary tool rather than a substitute for human expertise in professional examinations. The LDEK, like the LEK, requires not only factual knowledge but also the application of this knowledge in clinical contexts. Human candidates currently excel in these areas, as evidenced by high test scores. AI’s strong performance in factual recall highlights its potential, but its limitations in clinical reasoning underscore the need for ongoing development and refinement. Future AI advancements may narrow this gap, but for now, human cognition, especially in complex, applied scenarios, remains superior.

It is noteworthy to mention the limitations of this study which is a relatively small number of questions from each medical field that make statistical analysis less reliable and asking the ChatGPT in Polish language.

## Conclusions

ChatGPT’s performance on the LDEK demonstrates its potential as an educational tool in dentistry. However, the gap between AI and top human candidates underscores existing limitations in replicating human clinical reasoning and decision-making.

The lower motivation observed in LDEK takers compared to LEK candidates may reflect differences in career trajectories and training pathways in the fields of medicine and dentistry.
